# Gestational Chronodisruption Impairs Hippocampal Expression of NMDA Receptor Subunits Grin1b/Grin3a and Spatial Memory in the Adult Offspring

**DOI:** 10.1371/journal.pone.0091313

**Published:** 2014-03-24

**Authors:** Nelson Vilches, Carlos Spichiger, Natalia Mendez, Lorena Abarzua-Catalan, Hugo A. Galdames, David G. Hazlerigg, Hans G. Richter, Claudia Torres-Farfan

**Affiliations:** 1 Laboratorio de Cronobiologia del Desarrollo, Instituto de Anatomia, Histologia y Patologia, Facultad de Medicina, Universidad Austral de Chile, Valdivia, Chile; 2 Institute of Biological and Environmental Sciences, Zoology Building, Tillydrone Avenue, University of Aberdeen, Aberdeen, United Kingdom; Karlsruhe Institute of Technology, Germany

## Abstract

Epidemiological and experimental evidence correlates adverse intrauterine conditions with the onset of disease later in life. For a fetus to achieve a successful transition to extrauterine life, a myriad of temporally integrated humoral/biophysical signals must be accurately provided by the mother. We and others have shown the existence of daily rhythms in the fetus, with peripheral clocks being entrained by maternal cues, such as transplacental melatonin signaling. Among developing tissues, the fetal hippocampus is a key structure for learning and memory processing that may be anticipated as a sensitive target of gestational chronodisruption. Here, we used pregnant rats exposed to constant light treated with or without melatonin as a model of gestational chronodisruption, to investigate effects on the putative fetal hippocampus clock, as well as on adult offspring’s rhythms, endocrine and spatial memory outcomes. The hippocampus of fetuses gestated under light:dark photoperiod (12:12 LD) displayed daily oscillatory expression of the clock genes *Bmal1* and *Per2*, clock-controlled genes *Mtnr1b*, *Slc2a4*, *Nr3c1* and *NMDA* receptor subunits 1B-3A-3B. In contrast, in the hippocampus of fetuses gestated under constant light (LL), these oscillations were suppressed. In the adult LL offspring (reared in LD during postpartum), we observed complete lack of day/night differences in plasma melatonin and decreased day/night differences in plasma corticosterone. In the adult LL offspring, overall hippocampal day/night difference of gene expression was decreased, which was accompanied by a significant deficit of spatial memory. Notably, maternal melatonin replacement to dams subjected to gestational chronodisruption prevented the effects observed in both, LL fetuses and adult LL offspring. Collectively, the present data point to adverse effects of gestational chronodisruption on long-term cognitive function; raising challenging questions about the consequences of shift work during pregnancy. The present study also supports that developmental plasticity in response to photoperiodic cues may be modulated by maternal melatonin.

## Introduction

Day/night alternation provides a persistent cue to which virtually all organisms have adapted through the circadian system. Like adult individuals, developing fetuses require complex integrated molecular and physiological systems allowing adaptation to internal and external environmental time cues, in order to achieve an internal temporal order [1], [2]. In this context, melatonin has been thoroughly investigated as the main circulating circadian synchronizer. During pregnancy, the maternal pineal gland produces melatonin in a circadian fashion, with high plasma levels at nighttime and very low levels at daytime. Melatonin has the ability to cross all physiological barriers, including the placenta [3] and blood-brain barrier. Thus the fetus, in which the pineal does not synthesize melatonin, is exposed to the maternal melatonin rhythm, and hence indirectly to light:dark (LD) information [4], [5]. Notably, when pregnant dams are exposed to constant light at night, plasma melatonin is suppressed in both, diurnal and nocturnal animals [6], [7]. Light at night is a strong chronodisruptor, inducing significant derangement of the temporal organization of endocrinology, physiology, metabolism and behavior [8]. Hence, we hypothesize that gestational chronodisruption targets not only the maternal circadian system directly, but also the fetal circadian system indirectly; with the latter being probably driven by maternal melatonin.

In humans and animal models, several studies using clock gene mutants have demonstrated that chronodisruption has deleterious effects in physiology, including decreased performance in a variety of behavior and learning tests [9]–[14]. These observations have led to the recognition of the hippocampus as an important target of photoperiod disruption, since it is a strong peripheral oscillator in adult animals [12], [14]–[16]. Concerning development, there is compelling evidence that the fetal hippocampus is a crucial target of fetal programming [13], [17]–[19]. Therefore, the fetal hippocampus may be a sensitive target for deleterious signals derived from an inadequate maternal environment that could induce long-term effects on cognitive function [17], [20]–[23]. Although results from our group support the existence of daily rhythms in rat fetuses [2], [24], [25], it is presently unknown whether the hippocampus is a peripheral oscillator during fetal life; let alone the potential role of the maternal circadian system and melatonin on hippocampus’ development and adult functionality. Here we explored *in vivo* whether the fetal hippocampus possess daily rhythms of mRNA expression and if these rhythms are potentially entrained by maternal melatonin. We selected 18 days of gestation for sampling, to ensure an appropriate dissection of the hippocampus. As well, at this gestational age the fetal suprachiasmatic nucleus (SCN) does not display metabolic circadian rhythm yet [26], [27]. In the fetal hippocampus, we measured the expression of the canonical clock genes period 2 (*Per2),* brain and muscle aryl hydrocarbon receptor nuclear translocator like protein 1 (*Bmal-1*); melatonin receptors 1 and 2 *(Mtnr1a* and *Mtnr1b*); facilitated glucose transporter 4 (*Slc2a4*); glucocorticoid receptor (*Nr3c1*); and subunits of NMDA receptor (*Grin1B*, *Grin3A* and *Grin3B*) every 4 hours for 24-h. *Per2* and *Bmal1* are two clockwork core elements that in general are expressed in antiphase in functional circadian clocks, thus providing a broad picture of rhythmic clock gene expression [2], [24], [25]; *Mtnr1a* and *Mtnr1b*, *Slc2a4* and *Nr3c1*, are four genes directly or indirectly driven by the circadian system (clock-controlled genes) [28]–[30]. Further consideration to measure *Mtnr1a* and *Mtnr1b* expression was that direct actions of melatonin have been reported in the adult hippocampus [31], [32]. Finally, the aim to evaluate NMDA receptor subunits expression was two-fold: (1) to verify whether they undergo daily oscillation in the fetal hippocampus, as reported previously in adult and fetal SCN [33], and (2) as biomarkers of potential alteration in the mechanisms of memory, in which NMDA receptors play a key role [34], [35].

In addition, we explored whether gestational chronodisruption -secondary to maternal exposure to constant light-, modifies the daily expression of clock and clock-controlled genes in the fetal hippocampus; and also if this translates into long-term effects on the hippocampus’ function in the adult offspring. Finally, we explored whether melatonin supplementation to pregnant dams subjected to gestational chronodisruption may prevent the effects on the fetal and adult hippocampus.

## Material and Methods

### Animals

Animal handling and care was performed following the Guide for the Care and Use of Laboratory Animals of the Institute for Laboratory Animal Research of the National Research Council, relating to the conduct of ethical research on laboratory and other animals. The protocols were approved by the Bioethics Commission from Facultad de Medicina, Universidad de Chile (CBA#0234).

Timed-pregnant female Sprague-Dawley rats were obtained after mating (the day in which spermatozoa were observed in the smear of the vaginal contents was considered embryonic day 0) from Bioterio Central, Facultad de Medicina, Universidad de Chile. The dams were maintained in a 12∶12 light/dark cycle (lights on at 0700; about 400 lux at the head level, standard photoperiod in our colony) under controlled temperature (18–20°C), with food and water *ad libitum*.

At 10 days of gestation pregnant females were randomly separated in three groups (n  =  40 for each group), and kept under the following conditions until either 18 days of gestation (fetal protocols) or until delivery (long-term protocols):

LD: Control photoperiod 12:12 (12 h light/12 h dark).

LL: Constant light (24 h light).

LL+Mel: LL receiving 2μg/mL melatonin in drinking water between 1900 and 0700 hours.

In the 3 groups of pregnant females we measured water consumption between 1900–0700 h. Briefly, a bottle of water with or without melatonin was replaced from 10 days of gestation until 18 days of gestation or delivery. The bottles were weighed before and after the change at 1900 and at 0700 h, respectively. We did not find differences in water consumption between the groups (LD: 32.8±0.7; LL: 32.7±0.9; LL+Mel: 32.9±0.9 ml). In this regards, the total consumption of melatonin in LL+Mel females was 65.4±1.7 μg through 1900–0700 h (about 160 μg/kg of weight). Additionally, we previously reported that these doses of melatonin in drinking water translated into plasma levels close to those found under control conditions. All fetuses used in the present study were obtained and their tissues were either preserved or processed as described below, from larger cohorts raised in our previous report [2].

### Fetal hippocampus collection

Pregnant rats were euthanized with sodium thiopental (150 mg/kg) every 4 hours around the clock (five mothers per clock time, n = 30 mothers per group) and a maternal blood sample was collected as reported previously [2]. The fetuses were obtained via midline incision and the fetal brain was collected and stored in RNAlater to allow dissection of the hippocampus 24 h later, using a protocol reported previously [36]. After dissection, the hippocampus samples were kept in lysis buffer (SV Total RNA Isolation System, Promega Corp, WI, USA) until RNA extraction.

### Adult offspring protocols

The remaining pregnant females (n  =  10 per group) were allowed to deliver, when the mothers and their offspring were immediately returned to LD photoperiod (i.e., 12 h light/12 h dark; lights on at 0700; about 400 lux at the head level). Infants were weaned at 21 days old, with the males being raised to be studied at 90 days of age. These males for long-term protocols were housed in pairs (brothers together in standard cages of 48×27×20 cm), under LD cycle and controlled temperature (18–20°C), with food and water *ad libitum.*

### Long-term experiments

To evaluate the effects of the gestational treatments on memory in the adult offspring, we used the Morris Water Maze test [37], which measures spatial memory in rats. Briefly, male rats had to seek for a platform submerged in water (approximately 2 cm), which cannot be seen, smelled or heard, but found only on the basis of a fixed array of references that are located outside the pool. Thus, four start locations were randomly used N, S, E and W per day (with an interval of 15–30 min between trials). The animals were introduced in the pool and the time spent to find the platform (latency; the time necessary to reach to the platform) was measured from day 1 until day 5, and mean time per day (four trials) was used. This test was repeated daily along 5 consecutive days; one group (animals from one mother) was studied in the morning (between 0800–1000 h; n = 10 per gestational treatment) and a second group was studied in the afternoon (at 1700–1900 h; n = 10 per gestational treatment).

### Glucose tolerance

One week after finishing the water maze test, rats were subjected to an intraperitoneal glucose tolerance test (n = 20/maternal treatment, 2 male/litter). Briefly, the animals were deeply anesthetized using ketamine (0.3 ml 10%) xylazine (0.1 ml 2%) after 12 h fasting (the tests were performed at 0800 h), a blood drop was collected from the tail to measure plasma glucose levels (Accu-Chek; Roche) at –30, –15 and 0 min time points. Immediately after time 0, an injection of glucose in physiological serum (i.p. 1 g/kg) was given and glucose was measured in a blood drop at 15, 30, 45, 60, 90 and 120 min after injection.

### Adult hippocampus collection and plasma hormone assays

One week after the IP glucose tolerance tests were run, we collected the adult hippocampus from these animals. To this end, 10 animals from each gestational treatment were euthanized at 11 or 23 h (one male/litter) through overdose of thiopental as described above, and a blood sample was taken for subsequent measurement of glucose as described above and of melatonin and corticosterone as previously described by us [2]. The time points chosen to study day/night differences were selected taking into account our previous experience relative to the acrophase of the circadian rhythm of corticosterone and melatonin in this species.

Different tissues were dissected out and preserved in a tissue bank. The whole brain was stored in RNAlater for subsequent dissection of the hippocampus and maintained in lysis buffer afterwards, until RNA extraction (see details above).

### Quantitative RT-PCR (RT-qPCR)

Total RNA samples were isolated from fetal and adult hippocampus using SV Total RNA Isolation System according to the manufacturer’s instructions. This kit includes a digestion step with DNAse to rule out contamination with genomic DNA. About 1.0 µg of total RNA was then reverse-transcribed and the expression level of the mRNAs encoding for *Per2* (period 2), *Bmal-1* (brain and muscle aryl hydrocarbon receptor nuclear translocator like protein 1), *Mtnr1a* (melatonin receptor isoform 1), *Mtnr1b* (melatonin receptor isoform 2) and the housekeeping gene 18S-rRNA, were measured by qPCR using the methodology and primers previously described by us [24]. To measure the expression of the mRNA of facilitated glucose transporter 4 (*Slc2a4*), glucocorticoid receptor (*Nr3c1*), and NMDA receptor subunits (*Grin1b, Grin3a* and *Grin3b*), we used qPCR primers described by others (designed to span at least one intron; see **Table 1**). First, we confirmed the identity of the PCR products using semi-quantitative PCR (sq-PCR) for *Slc2a4*, *Nr3c1* and NMDA receptor subunits, which were amplified in the calibrator sample (adult hippocampus cDNA pool). The sq-PCR products were purified by chromatography (DNA Wizard PCR Preps; Promega) and sequenced. The similarity degree of each sq-PCR product with the corresponding rat gene sequence reported in GeneBank, was determined using the BLASTN 2.2.1 tool (www.ncbi.nlm.nih.gov). Percentage identities were 98% for *Slc2a4*, 98% for *Nr3c1,* 100% for *Grin1b,* 97% for *Grin3* and 96% for *Grin3b*.

**Table 1:** RT-qPCR primers for genes studied**.**

Gene	Primer sequence (sense/antisense)	Size (bp)	Annealing T° (°C)	*E*
*Per2^a^*	caccctgaaaagaaagtgcga/	148	62	2.01
	caacgccaaggagctcaagt			
*Bmal^a^*	ccgatgacgaactgaaacacct/	215	64	2.05
	tgcagtgtccgaggaagatagc			
*Mtnr1a^a^*	tttactatcgtggtggacatcc/	206	60	2.02
	gcactaacttgacaatgcagatatc			
*Mtnr1b^a^*	ctcactctggtggccttgg/	250	60	1.93
	aactgcgcaggtcactgg			
*Nr3c1* ^a^	caaagccgtttcactgtccatg/	313	60	2.04
	caatttcacactgcctccgttg			
*Slc2a4* ^b^	cccccgatacctctacatcatc/	90	64	2.04
	gcatcagacacatcagcccag			
*Grin1b* ^c^	gaatgatgggcgagctactca/	71	60	2.20
	acgctcattgttgatggtcagt			
*Grin3a* ^c^	catcaaacccccaaaatgct/	75	60	2.18
	gaaaggcaaaacatacagaaaatgg			
*Grin3b* ^c^	tcagtagcatggcccttgtaca/	87	60	2.29
	cctcagatccgcctgttttc			
*18S-rRNA^a^*	gtaacccgttgaaccccatt/	150	60	2.02
	ccatccaatcggtagtagcg			

E: Efficiency assay using fetal and adult hippocampus pooled RNA. a: [24]; b: [59]; c:[60].

Relative amounts of all mRNAs were calculated by the comparative ΔΔCt method using the 2^−ΔΔCt^ equation. To validate the quantification using the ΔΔCt method for *Slc2a4*, *Nr3c1* and NMDA receptor subunits, a dilution curve (range 0.25–50 ng input total RNA) was performed in the calibrator (adult hippocampus cDNA pool) and in a pool of fetal hippocampus. The Ct obtained versus RNA concentration was plotted and the slope (m) of the lineal regression was calculated, with the efficiency (*E*) in each curve for each gene being determined as *E* = 10^−(1/m*)*^. Efficiencies ranged between 2.01–2.10 (equivalent 101–110 % respectively; see **Table 1**). The first ΔCt is the difference in the sample of the Ct values between the gene of interest and its respective 18S-rRNA, while the second ΔCt is the difference in the calibrator sample between the gene of interest and its respective 18S-rRNA. The sample and the calibrator were assayed simultaneously. All the samples were amplified in duplicate in at least three mRNA concentrations (range 2.5–100 ng). A melting curve analysis was performed on each sample after the final cycle to ensure that a single product was obtained, and agarose gel electrophoresis confirmed that the single PCR product was of the expected size.

### Statistical analysis

Data are expressed as Mean ± SEM. In the fetal hippocampus the 24 h daily oscillation of 2^−ΔΔCt^ for each gene measured, were analyzed by one way ANOVA using Newman-Keuls as post hoc test. The LD, LL and LL+Mel 24 h changes in the values of 2^−ΔΔCt^ for each gene were analyzed by two way ANOVA using Bonferroni as a post hoc test. Mean data were fitted to a theoretical cosine function, and acrophases were calculated for each gene. Acrophases of genes showing significant rhythm (present in LD and LL+Mel hippocampus) were compared by Student t-test. Fetal and postnatal weights were analyzed using one way ANOVA and Newman-Keuls as post hoc test. The day/night differences of plasma melatonin and corticosterone and of 2^−ΔΔCt^ for each gene, were analyzed by Two way ANOVA using Bonferroni as post hoc test. Results were considered significant when P<0.05. Statistical analyses were performed using GraphPad Prism software (version 3.02; GraphPad Software Inc., San Diego, CA).

## Results

**Figure 1:**
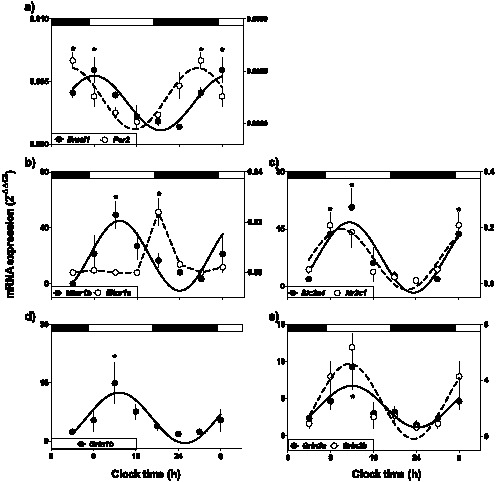
Daily oscillatory expression of *Per2* and *Bmal1* (a); *Mtnr1a* and *Mtnr1b* (b); *Nr3c1* and *Slc2a4* (c); NMDA receptor subunit 1B [*Grin1b*] (d); and 3A-3B [*Grin3a* and *Grin3b*] (e), in the fetal rat hippocampus at 18 days of gestational age from pregnant dams maintained in 12:12 LD photoperiod. Mean ± SEM of 2^−ΔΔCt^. *: Different from other time points (P<0.05; ANOVA). Black bars indicate lights-off hours. In each graph the value at 0400 h is repeated in the next 24 h.

In the fetal hippocampus under control photoperiod (12∶12 light:dark; LD), the canonical clock genes *Per2* and *Bmal1* were transcribed with a daily rhythm (P<0.05; by ANOVA and Newman-Keuls; **Fig. 1a**), with acrophases occurring during dark period and at the beginning of the light hours, respectively. In addition, several genes driven by the circadian system, such as *Mtnr1a* and *Mtnr1b* (melatonin receptor isoforms), *Nr3c1* (glucocorticoid receptor) and *Slc2a4* (glucose transporter 4) displayed daily rhythms of expression (P<0.05; by ANOVA and Newman-Keuls; **Fig. 1b-c**); with acrophases at the beginning of the light hours (**Table 2**). The subunits *Grin1b*, *Grin3a* and *Grin3b* of the N-methyl D-aspartate receptor (NMDAR) were also expressed with a daily rhythm (P<0.05; by ANOVA and Newman-Keuls; **Fig. 1d-e**), displaying acrophases at early day (**Table 2**).

**Table 2.Effects:** of melatonin supplementation to pregnant dams kept under constant light on the circadian rhythm of clock and clock-controlled genes in the fetal hippocampus (Mean ± SEM).

	LD			LL + Mel		
	Acrophase (h)	Mesor	R^2^	Acrophase	Mesor	R^2^
		(2^−ΔΔCt^/24)		(h)	(2^−ΔΔCt^/24)	
***Bmal1***	8.18±0.29	0.0032±0.0007	0.69	17.70±0.19*	0.0039±0.0004	0.62
***Per2***	3.45±0.37	0.0012±0.0005	0.67	12.48±0.12*	0.0021±0.0003	0.51
***Mtnr1b***	13.92±0.78	20.39±6.98	0.54	24.28±0.13*	24.98±2.70	0.61
***Mtnr1a***	19.95±0.49	0.0047±0.0020	NS	N.D.	N.D.	N.D.
***Slc2a4***	11.35±0.33	7.78±3.17	0.55	19.62±0.11*	3.23±0.59	0.59
***Nr3c1***	10.43±1.10	0.0868±0.0350	0.61	14.63±0.12*	0.0037±0.0008 *	0.72
***Grin1b***	12.30±0.32	5.14±0.88	0.54	15.05±0.16*	3.54±0.49	0.50
***Grin3b***	12.05±0.44	4.78±2.85	0.60	19.82±0.14*	1.46±0.72	0.58
***Grin3a***	11.92±0.31	7.54±1.19	0.55	20.08±0.17*	3.22±0.62*	0.58

N.D.: Not detectable. *: Different to LD (student t test).

**Figure 2:**
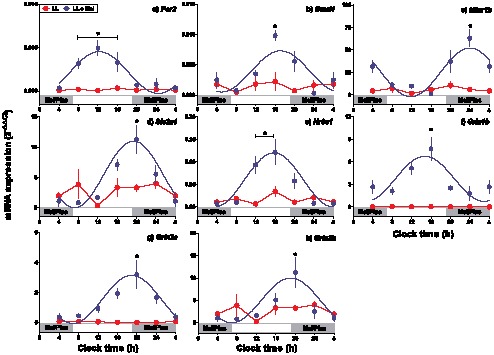
Effect of gestational chronodisruption and maternal melatonin replacement on daily oscillatory transcription of *Per2* (a), *Bmal1* (b), *Mtnr1b* (c), *Slc2a4* (d), *Nr3c1* (e) and NMDA receptors (*Grin*) subunits (f-h) in the fetal rat hippocampus at day 18 of gestation, from pregnant dams under LL (*Red symbols*) and LL + Mel (*Blue symbols*). Mean ± SEM of 2^−ΔΔCt^ *: Different from other time points (P<0.05; ANOVA). Note that LL was different to LL + Mel (P<0.05; Two way ANOVA). Grey bars indicate the clock time of melatonin replacement or placebo. In each graph the value at 0400 h is repeated in the next 24 h.

Gestational chronodisruption (maternal exposure to constant light; LL), suppressed the daily rhythm of expression for all genes studied in the fetal hippocampus (P<0.05 by ANOVA and Newman-Keuls; **Fig. 2a-h**; red symbols); while mean levels of expression were reduced. In the fetal hippocampus from mothers in LL but supplemented with melatonin between 1900 and 0700 h (LL+Mel), we observed daily rhythms of gene expression (P<0.05; by ANOVA and Newman-Keuls; **Fig. 2a-h** **;** blue symbols); displaying a phase delay of about 8–10 hours, except for *Nr3c1* and *Grin1b* (which were delayed by about 4-h; **Table 2**), compared with control LD fetuses. Meanwhile, the mRNA encoding for *Mtnr1a* was undetectable in the hippocampus under LL conditions and therefore it was not included in Fig. 2.

**Figure 3:**
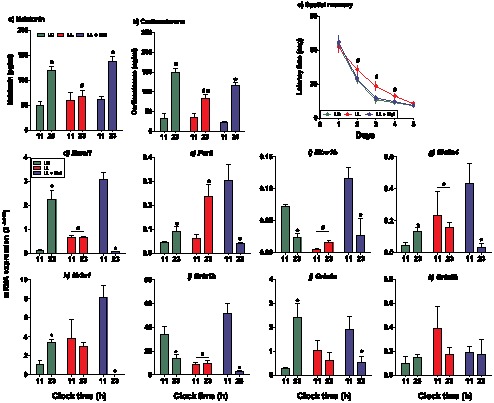
Long-term (postnatal day 90) effects of maternal exposure to constant light and maternal melatonin replacement on plasma melatonin (a) and corticosterone (b), spatial memory (c) and on day/night differences in expression of clock genes (d-e), clock controlled genes (f-h) and NMDA receptor subunits (i-k). Mean ± SEM for plasma hormones, latency time and of 2^−ΔΔCt^. 90 days old offspring gestated under LD (*Green symbols*), LL (*Red symbols*) and LL + Mel (*Blue symbols*). #: Different to LD and LL+Mel (P<0.05; Two way ANOVA). *: Different to 11-h (P<0.05; Two way ANOVA).

In the adult offspring (at 90 days of age) gestated under constant light and raised in LD conditions after birth, we observed both, significant lack of day/night differences in plasma melatonin and reduced day/night differences in corticosterone levels, relative to either LD or LL+Mel adult offspring (P<0.05; Two way ANOVA **Fig. 3a and 3b**; respectively). In adult LL offspring we found higher basal glucose and impaired fasted glucose tolerance (**Figure S1a** and **1b**; respectively); whereas the adult LL+Mel offspring showed an improvement of the response to I.P. glucose, reaching a response pattern similar to the adult LD offspring (**Figure S1b**). Despite these persistent hormonal and metabolic changes, there were no differences in body weight between LD, LL and LL+Mel animals neither at birth, 30 nor 90 days of postnatal age (**Table 3**).

**Table 3:** Effects of gestational condition (LD, LL and LL+Mel) on body weight (g) at birth, 30 and 90 days of age (Mean ± SEM).

Age	LD	LL	LL + Mel
At birth (n = 20)	6.26±0.21	6.13±0.26	6.12±0.39
30 days (n = 20)	136.80±3.63	145.00±5.02	149.14±8.17
90 days (n = 20)	559.30±22.44	552.50±17.38	569.0±10.65

In every experimental group we performed Morris water maze tests at two different clock times. Given that no differences in time latency were found between morning and evening testing, we grouped all the analysis in one experimental set (n = 20). Adult LL animals displayed a longer latency time along consecutive days in Morris Water Maze test (P<0.05; Two way ANOVA; **Fig. 3c**), and a marked change in day/night expression level of *Bmal1*, *Per2*, *Mtnr1b*, *Slc2a4*, *Nr3c1* and subunits of NMDA receptors in the hippocampus (P<0.05; Two way ANOVA**;** **Fig 3d-j**) relative to LD conditions. In contrast, the adult offspring from LL mothers that received melatonin (LL+Mel) showed day/night differences in corticosterone and melatonin plasma levels (P<0.05; Two way ANOVA; **Fig. 3a and 3b** **),** day/night differences for all genes studied in the hippocampus (P<0.05; Two way ANOVA; **Fig. 3d-j**) but not for *Grin3b* (**Fig. 3k**), as well as a latency time in the Morris Water Maze test similar to that found in adult LD animals (**Fig. 3c**).

## Discussion

At 18 days gestation the clock genes *Bmal1* and *Per2* were expressed with a daily rhythm in the fetal rat hippocampus. Consistent with the fact that this organ is a peripheral clock already in fetal life, we also found that four genes direct or indirectly driven by the circadian system such as *Mtnr1a, Mtnr1b, Nr3c1* and *Slc2a4* [28]–[30] and NMDA receptor subunits *Grin1b, Grin3a* and *Grin3b* displayed daily rhythms of expression. The acrophase of these genes, studied as clock outputs, occurred during the light phase, delayed by about 6 hours with that of the fetal corticosterone rhythm (at 0600 h; [24]) and close to the maximum expression of *Bmal1* found here in fetal and in adult rodent hippocampus [14], [38]. Similar to results reported by Jilg et al [14], in the hippocampus we found that the mRNA expression of *Bmal1* and *Per2* did not display an anti-phase, in contrast with observations in other peripheral clocks [1], [39]. Despite the lack of anti-phasic patterns on gene expression in fetal or in adult rodent hippocampus (present report and [14]; respectively), it has been demonstrated that the hippocampus’ circadian clockwork is critical in memory formation and consolidation, using both knockout models and lesions of the suprachiasmatic nucleus [11], [12], [14]. Therefore, the present results support that a synchronized clock may operate in the fetal hippocampus. Interestingly, in the adult hippocampus we found that the mRNA expression of clock and clock-controlled genes, were maximal during the light hours; as observed in the fetus. Therefore, independent of developmental stage, the hippocampus rhythms could be synchronized by a similar signal during fetal and adult life. However, there is little information about signals contributing to hippocampus’ clock synchronization, either in the fetus or adult.

In other models, melatonin and glucocorticoid rhythms have been suggested as signals involved in memory and clock synchronization in the hippocampus (reviewed in [12], [40]. In line with this, in the fetal hippocampus we found a daily oscillatory expression of both melatonin receptors (*Mtnr1a and Mtnr1b*; with *Mtnr1b* displaying the higher expression level) and of glucocorticoid receptor (*Nr3c1*), supporting that maternal melatonin and/or fetal glucocorticoids may act as potential signals that synchronize the putative fetal rat hippocampus circadian clock. In agreement with this idea, we found that maternal exposure to constant light, that induced suppression of both maternal plasma melatonin and fetal corticosterone rhythm, resulted in the absence of rhythms in expression of the clock genes *Bmal1* and *Per2* in the fetal hippocampus as reported in fetal adrenal [2]. From these experiments, we cannot distinguish whether in LL fetuses an intrinsic rhythm is present, but desynchronized between individual fetuses, or whether maternal chronodisruption altered the fetal hippocampus circadian clock. In this context, we observed that the fetal hippocampus from LL pregnant dams that received melatonin supplementation between 1900 and 0700 h, displayed a daily rhythm in both clock and clock-controlled genes. However, we observed that the daily rhythms of gene expression displayed a phase delay of about 8–10 h (except for *Nr3c1* and *Grin1b*, which were delayed by 4 h versus control LD fetuses), similar to our previous results in the fetal rat adrenal [2]. These findings support that the fetal hippocampus daily rhythms are potentially synchronized by maternal melatonin, since a delay of 8 h was also found for melatonin circadian rhythm between control and LL+Mel mothers [2]. Melatonin acts to provide a time signal to several organs during fetal and adult life [41], [42], nevertheless it is important keep in mind that another mechanism could be operating in the fetal hippocampus, and melatonin could be inducing oscillation directly in this tissue as reported previously in other organs like *pars tuberalis* [42]–[44]. Another maternal signal involved in the synchronization of the fetal hippocampus clock might be maternal corticosterone circadian rhythm. However, only a small portion of corticosterone crosses the placenta [45], while our results showed that the maternal circadian rhythm of corticosterone was not altered by constant light along pregnancy, with only a slight phase delay occurring for peak maternal plasma corticosterone in LL relative to LD control mothers [2]. Therefore, in our hands it is unlikely that maternal corticosterone may act as a synchronizing signal, at least for fetal hippocampus. Nonetheless, we cannot exclude that the effects observed on the fetal hippocampus circadian clock, may arise from an effect of circadian rhythm changes in maternal melatonin combined with lower fetal plasma corticosterone. Certainly, more studies are required to fully establish the role of maternal melatonin and fetal corticosterone rhythms on fetal circadian rhythms synchronization, as well as the potential role of other maternal signals such as maternal food entrainment [46].

Given that maternal chronodisruption is able to alter circadian rhythms and other aspects of fetal and maternal physiology [2], [47], [48], we hypothesized that maternal chronodisruption may contribute to the onset of adult disease, as it has been abundantly reported for prenatal adverse conditions such as undernutrition and hypoxia [49]–[51]. In this context, we found that adult offspring which had been gestated under constant light exhibited persistent differences already observed during fetal life, namely: low levels of nighttime plasma corticosterone, lack of day/night differences in clock and clock-controlled gene expression accompanied by an overall decrease in expression of NMDA receptor subunits in the hippocampus; as well as a significant deficit of spatial memory. In addition, the adult LL offspring did not display day/night differences in plasma melatonin, supporting that prenatal exposure to constant light induced a permanent effect on both, the circadian and endocrine systems. Interestingly, in adult LL offspring we found altered plasma glucose homeostasis with higher basal glucose and impaired fasted glucose tolerance; as reported in a rat model of chronodisruption imposed by chronic photoperiod phase shift throughout pregnancy [52]. Taken together, all these findings are in agreement with other models of fetal programming of adult disease, like maternal undernutrition and hypoxia [49]–[51]. Therefore, both gestational chronodisruption challenges, chronic photoperiod phase shift [52] and constant light (present report; [2]), provide evidence suggesting that alterations of the photoperiod during gestation may persistently alter different postnatal physiological outcomes.

Exposure to constant light along pregnancy suppresses the maternal melatonin rhythm, interrupting melatonin passage from mother to fetus [2]. Maternal melatonin may synchronize fetal circadian rhythms and also directly regulate different fetal physiological functions (revised in [25]). Therefore, we anticipated that restoration of the maternal plasma melatonin rhythm may prevent the long-term effects observed in the progeny. In this context, the adult offspring from mothers exposed to constant light that received melatonin, showed an improvement in glucose tolerance curves and also in the spatial memory task, reaching values similar to those measured in control animals. Interestingly, we found that hippocampal day/night differences in clockwork and NMDA receptor subunits gene expression, which were absent in the LL offspring, were present in the adult progeny from LL+Mel mothers, albeit with a pattern almost opposite to that found in control LD offspring. Although we did not explore the expression of these genes along the whole 24 h, measurement of day/night differences at least give an approximate indication of the circadian system being altered in the hippocampus; in turn probably altering the molecular mechanisms of spatial memory, as described in knockout clock gene models [14]. The ability to restore day/night differences in adult offspring which had been gestated under LL+Mel conditions, together with the differences found in the fetal hippocampus, support a role of the maternal melatonin circadian rhythm in generating these rhythms in the growing fetus, and probably in contributing to the long-term stability of these rhythms until adult life.

There is limited evidence about the mechanisms of action of melatonin in the hippocampus; however, it has been suggested that melatonin might act as a signal gathering hippocampal function through a mechanism involving the activation of NMDA receptors [53]–[55]. Regardless of the mechanisms involved, there is compelling evidence implicating *Mtnr1b* (isoform 2 of melatonin receptor) in learning and memory processing. Relative to wild-type mice, *Mtnr1b* knockout mice submitted to an elevated plus-maze on two consecutive days did not show shorter transfer latencies to enter a closed arm on the second testing day [31]. Meanwhile, *in vitro* studies in hippocampus slices demonstrated that melatonin inhibits long-term potentiation (LTP) through a mechanism that involved *Mtnr1b* receptor activation [56]. Using electrophysiological recording on hippocampal field CA1 slices, these authors demonstrated that theta burst stimulation induced attenuated LTP in *Mtnr1b* knockouts relative to wild-type mice. These combined results are particularly relevant in the context of the present investigation, given that in the fetal hippocampus under LL conditions, we found that the *Mtnr1a* mRNA was undetectable while the *Mtnr1b* mRNA was markedly downregulated. Moreover, in the hippocampus from adult offspring which had been gestated under LL conditions, we also found significantly decreased levels of *Mtnr1b* mRNA relative to both, LD and LL+Mel adult offspring.

Since rhythmic melatonin synthesis by the rat pineal does not begin until 5–10 days post-partum [57], [58], the present data support that circadian rhythmicity in the fetal hippocampus is synchronized by maternal plasma melatonin acting as a transplacental signal. Overall, our findings suggest that the hippocampus circadian clock was entrained *in utero* and remained programmed through adulthood. In turn, the early life influence of chronodisruption and the ensuing lack of melatonin may have impaired molecular mechanisms of hippocampal spatial memory consolidation, as described in other models [12], [14].

Collectively, the present results provide the first evidence pointing to adverse effects of gestational chronodisruption on long-term cognitive function; raising challenging questions about the consequences of shift work during pregnancy. As a chronotherapeutic, melatonin appears to be most effective in contexts where normal light-dependent inputs are lacking. The present study extends this concept to the domain of developmental plasticity in response to photoperiodic cues, which remains largely under-studied in our lights-on society.

## Supporting Information

Figure S1 Long-term effects of gestational chronodisruption and maternal melatonin replacement on day/night glucose basal levels (a) and response to i.p. glucose (b) in adult rats. Mean ± SEM. 90 days old offspring gestated under LL (*red*), LL+Mel (*blue*) and LD (*green*). **#**: Different to LD and LL+Mel (P<0.05; Two way ANOVA); *: Different to basal glucose levels at 11-h (P<0.05; Two way ANOVA). **: Different to fasted glucose basal levels (P<0.05; ANOVA) for response to i.p. glucose; &: different to LD and LL+Mel (P<0.05; Two way ANOVA).
(DOCX)
