# Supplementary material for: Gestational Chronodisruption Impairs Hippocampal Expression of NMDA Receptor Subunits Grin1b/Grin3a and Spatial Memory in the Adult Offspring
Source: PLoS One. 2014 Mar 24;9(3):e91313. doi: 10.1371/journal.pone.0091313 (PMC3963867; doi:10.1371/journal.pone.0091313)
Supplement: Supplementary file 1 [file pone.0091313.s001.docx]

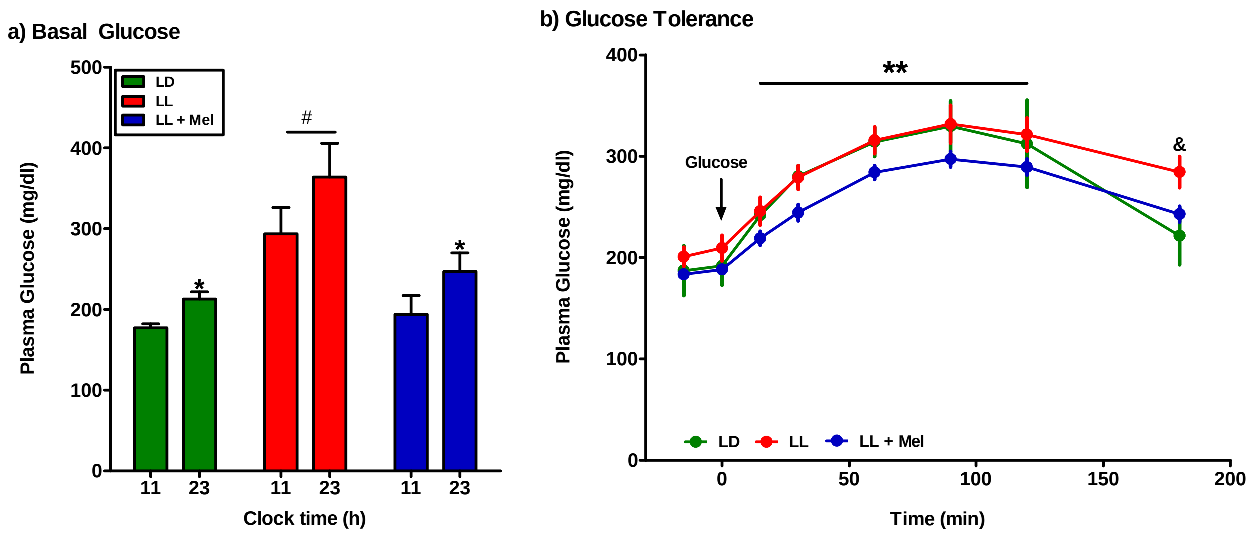
a) Basal Glucose
Plasma Glucose (mg/dl)
500
400
300
200
100
0
LD
LL
LL + Mel
#
*
*
11
23
11
23
11
23
Clock time (h)
b) Glucose Tolerance
Plasma Glucose (mg/dl)
400
300
200
100
0
**
Glucose
&
LD
LL
LL + Mel
0
50
100
150
200
Time (min)
